# Supplementary material for: Many-Body Second-Order Green’s Function Theory for Ab Initio Molecular Quantum Electrodynamics
Source: J Chem Theory Comput. 2026 Oct 28;22(21):11287–302. doi: 10.1021/acs.jctc.6c01285 (PMC13757762; doi:10.1021/acs.jctc.6c01285)
Supplement: Supplementary file 1 [file ct6c01285_si_001.pdf]

Supporting Information for

Many-Body Second Order Green's Function Theory for *Ab Initio* Molecular Quantum Electrodynamics

Amirhosein Amini[1], Jaime Cerda[2], Leopoldo Mejía[2,*], Arkajit Mandal[1,†]

[1]Department of Chemistry, Texas A&M University, College Station, Texas 77843, USA

[2]Departamento de Física y Astronomía, Universidad Andrés Bello, Santiago 837-0136, Chile

[*] leopoldo.mejia@unab.cl, [†] mandal@tamu.edu

Contents

1 Electronic GF2 **S2**
- 1.1 Non-orthogonal basis and finite-temperature notation . . . S2
- 1.2 One-particle Matsubara Greenś function . . . S2
- 1.3 Reduced density matrix from the Matsubara sum . . . S3
- 1.4 Mean-field reference Greenś function in a non-orthogonal basis . . . S3
- 1.5 Electronic GF2 self-energy . . . S4
- 1.6 Two-body expectation value and total energy . . . S5
- 1.7 Self-consistent electronic GF2 procedure . . . S5

2 QED-GF2 **S6**
- 2.1 Matsubara Green's Functions . . . S7
- 2.2 Non-interacting boson propagator . . . S8
- 2.3 Derivation of the electron-boson self-energy and polarization . . . S8
- 2.4 Dyson equations . . . S10
- 2.5 Total energy of the coupled electron-boson system . . . S10
- 2.6 Validation of the total-energy expression using an exactly solvable model . . . S14
- 2.7 CS-GF2 benchmark for the two-site Hubbard-Holstein model . . . S16
- 2.8 Practical self-consistency loop . . . S18
- 2.9 Projected Lang–Firsov GF2 (LF-GF2) . . . S18

3 Convergence Results **S25**
- 3.1 β Convergence . . . S25
- 3.2 Matsubara Grid Convergence . . . S25

4 Optimized Geometries **S26**

Note: This Supporting Information gives the algebraic details behind the electronic GF2 and QED-GF2 working equations used in the main text. The notation follows the manuscript unless stated otherwise. In particular, bold symbols denote matrices in the atomic-orbital basis, repeated orbital indices are summed, and finite-temperature quantities are evaluated in the grand canonical ensemble. The derivation of electronic GF2 is inspired by Refs. [1, 2, 3, 4].

1 Electronic GF2

1.1 Non-orthogonal basis and finite-temperature notation

In this document, Latin indices label atomic orbitals, and the spin indices are dropped due to considering a closed-shell system.

We start from a set of real non-orthogonal single-electron atomic-orbital states, $\varphi_i(r)$ and we introduce an overlap matrix $S_{ij} = \langle\varphi_i|\varphi_j\rangle$. Since we are working in a non-orthogonal basis, the anticommutation relation for creation and annihilation operators will no longer be $\{a_i^\dagger, a_j\} = \delta_{ij}$[5, 6], and instead can be written as

$$\{a_i^\dagger, a_j\} = (S^{-1})_{ji}. \tag{1.1}$$

The Hamiltonian for interacting electrons is expressed in terms of creation and annihilation operators

$$\hat{H} = \sum_{ij} h_{ij} a_i^\dagger a_j + \frac{1}{2}\sum_{ijkl} v_{ijkl} a_i^\dagger a_k^\dagger a_l a_j, \tag{1.2}$$

where h_{ij} and v_{ijkl} are the one- and two-electron integrals, respectively.

We work at finite temperature T and chemical potential μ in the **Grand Canonical Ensemble** with density operator

$$\hat{\rho} = \frac{e^{-\beta(\hat{H}-\mu\hat{N})}}{Z(\beta)} \tag{1.3}$$

where $\beta = \frac{1}{k_B T}$ and $Z(\beta) = \mathrm{Tr}[e^{-\beta\hat{K}}]$, $\hat{K} = \hat{H} - \mu\hat{N}$. In the non-orthogonal basis, the number operator is

$$\hat{N} = \sum_{ij} S_{ij} a_i^\dagger a_j. \tag{1.4}$$

The thermal expectation value of an operator $\hat{A}$ is defined as

$$\langle\hat{A}\rangle = \mathrm{Tr}\left[\hat{\rho}\hat{\mathrm{A}}\right] = Z^{-1}(\beta)\mathrm{Tr}\left[e^{-\beta\hat{K}}\hat{A}\right]. \tag{1.5}$$

The reduced density matrix is defined as

$$P_{ij} \equiv \sum_\sigma \langle a_{j\sigma}^\dagger a_{i\sigma}\rangle. \tag{1.6}$$

1.2 One-particle Matsubara Greenś function

In the grand-canonical ensemble at inverse temperature β and chemical potential μ, the thermal average of an operator $\hat{O}$ is

$$\langle\hat{O}\rangle = Z^{-1}\,\mathrm{Tr}\Big[e^{-\beta\hat{K}}\hat{O}\Big], \qquad \hat{K} \equiv \hat{H} - \mu\hat{N}, \qquad Z = \mathrm{Tr}\Big[e^{-\beta\hat{K}}\Big]. \tag{1.7}$$

We define the imaginary-time Heisenberg operators by

$$a_j(\tau) \equiv e^{\tau\hat{K}} a_j e^{-\tau\hat{K}}, \qquad a_k^\dagger(\tau) \equiv e^{\tau\hat{K}} a_k^\dagger e^{-\tau\hat{K}}, \qquad 0 \le \tau \le \beta. \tag{1.8}$$

The one-particle Matsubara Green's function is defined by

$$G_{jk}(\tau) \equiv -\left\langle \mathcal{T}_\tau \, a_j(\tau) \, a_k^\dagger(0) \right\rangle, \tag{1.9}$$

where $\mathcal{T}_\tau$ is the imaginary-time ordering operator. Taking the $\tau \to 0^-$ limit gives

$$G_{jk}(0^-) = \left\langle a_k^\dagger a_j \right\rangle, \tag{1.10}$$

so the one-particle reduced density matrix is $P_{jk} = 2G_{jk}(0^-)$ for the closed-shell system considered here.

Anti-periodicity and Matsubara frequencies. Using the KMS condition[7, 8, 9, 1], one shows that

$$G_{jk}(\tau) = -G_{jk}(\tau + \beta), \qquad -\beta < \tau < 0. \tag{1.11}$$

The anti-periodicity results in the *fermionic Matsubara frequencies*

$$\omega_n = (2n+1)\frac{\pi}{\beta}, \qquad n \in \mathbb{Z}. \tag{1.12}$$

Defining

$$G(i\omega_n) \equiv \int_0^\beta G(\tau) e^{i\omega_n \tau} \mathrm{d}\tau, \tag{1.13}$$

the inverse transform is

$$\boxed{G(\tau) = \frac{1}{\beta} \sum_{n=-\infty}^{\infty} G(i\omega_n) e^{-i\omega_n \tau}.} \tag{1.14}$$

For real $G(\tau)$ on $(0, \beta)$ this simplifies to

$$\boxed{G(\tau) = \frac{2}{\beta} \operatorname{Re} \sum_{n=0}^{\infty} G(i\omega_n) e^{-i\omega_n \tau}.} \tag{1.15}$$

1.3 Reduced density matrix from the Matsubara sum

From Eq. (1.9) one has $G_{jk}(0^-) = \langle a_k^\dagger a_j \rangle$. For a closed-shell electronic system, the spin-summed density matrix is

$$P_{kj} = \sum_\sigma \langle a_{j\sigma}^\dagger a_{k\sigma} \rangle = 2\langle a_j^\dagger a_k \rangle = 2G_{kj}(0^-). \tag{1.16}$$

Using the anti-periodicity Eq. (1.11) and the Fourier representation Eq. (1.15), one arrives at

$$\boxed{P_{kj} = 2G_{kj}(0^-) = -2G_{kj}(\beta^-) = \frac{4}{\beta} \operatorname{Re} \sum_{n=0}^{\infty} e^{-i\omega_n 0^-} G_{kj}(i\omega_n).} \tag{1.17}$$

1.4 Mean-field reference Greenś function in a non-orthogonal basis

The mean-field reference Hamiltonian $\hat{H}_0 = \sum_{ij} F_{ij} \, a_i^\dagger a_j$ is diagonalized by transforming to an orthogonal basis via $\mathbf{X}\mathbf{X}^T = \mathbf{S}^{-1}$, where $\overline{\mathbf{F}} \equiv \mathbf{X}^T \mathbf{F} \mathbf{X}$ is the Fock matrix in the orthogonal basis. Evaluating the Matsubara

Green's function in the eigenbasis and transforming back to the AO basis gives the mean-field reference Green's function in imaginary time:

$$\boxed{\mathbf{G}_0(\tau) = \mathbf{X}\, e^{-\tau(\overline{\mathbf{F}}-\mu\mathbf{I})}\left[\frac{\theta(-\tau)}{1+e^{\beta(\overline{\mathbf{F}}-\mu\mathbf{I})}} - \frac{\theta(\tau)}{1+e^{-\beta(\overline{\mathbf{F}}-\mu\mathbf{I})}}\right]\mathbf{X}^T.} \tag{1.18}$$

The Fourier transform of Eq. (1.18) yields the important frequency-domain result

$$\boxed{\mathbf{G}_0(i\omega_n) = \left[(\mu + i\omega_n)\,\mathbf{S} - \mathbf{F}\right]^{-1}.} \tag{1.19}$$

We define the self-energy through the inverse Dyson equation

$$\boldsymbol{\Sigma}(i\omega_n) \equiv \mathbf{G}_0^{-1}(i\omega_n) - \mathbf{G}^{-1}(i\omega_n), \tag{1.20}$$

which gives the interacting Dyson equation

$$\mathbf{G}(i\omega_n) = \left[(\mu + i\omega_n)\mathbf{S} - \mathbf{F} - \boldsymbol{\Sigma}(i\omega_n)\right]^{-1}. \tag{1.21}$$

1.5 Electronic GF2 self-energy

GF2 uses a Hartree-Fock ansatz as the reference Hamiltonian. The closed-shell Fock matrix is[10]

$$F_{ij} = h_{ij} + \frac{1}{2}P_{kl}\left(2v_{ijkl} - v_{ilkj}\right). \tag{1.22}$$

The Fock matrix is rebuilt using the correlated density matrix. The static first-order Hartree and Fock contributions are therefore already included in $\mathbf{F}$, while $\boldsymbol{\Sigma}$ in Eq. (1.21) contains only the beyond-Hartree-Fock correlation contribution.

The self-energy is approximated by the second-order direct and exchange skeleton diagrams. The residual interaction beyond the Hartree-Fock reference is the normal-ordered two-electron fluctuation operator

$$\boxed{\hat{W} = \frac{1}{2}\sum_{ijkl}\sum_{\sigma\sigma'} v_{ijkl} : a^\dagger_{i\sigma}a^\dagger_{k\sigma'}a_{l\sigma'}a_{j\sigma} : .} \tag{1.23}$$

The residual operator is normal ordered with respect to the mean-field reference. Its first-order self-energy contribution vanishes because the static Hartree and Fock terms have already been included in $\mathbf{F}$. Expanding the exact Green's function to second order via the S-matrix and applying Wick's theorem retaining only connected diagrams, the second-order Born self-energy in imaginary time is

$$\boxed{\Sigma^{ee,(2),0}_{ij}(3,4) = G^{(0)}_{kl}(3,4)G^{(0)}_{mn}(3,4)G^{(0)}_{pq}(4,3)v_{impk}\left(2v_{jnlq} - v_{jlnq}\right).} \tag{1.24}$$

The factor $2v_{jnlq}$ is the spin-summed direct (bubble) contribution and $-v_{jlnq}$ is the same-spin exchange contribution. Using imaginary-time translation invariance and the fermionic antiperiodicity $G(-\tau) = -G(\beta-\tau)$, this reduces to a single time variable. GF2 replaces the bare propagators by dressed self-consistent ones ($G^{(0)} \to G$), yielding the electronic GF2 self-energy:

$$\boxed{\Sigma^{ee}_{ij}(\tau) = -G_{kl}(\tau)G_{mn}(\tau)G_{pq}(\beta-\tau)v_{impk}\left(2v_{jnlq} - v_{jlnq}\right), \qquad 0 < \tau < \beta.} \tag{1.25}$$

1.6 Two-body expectation value and total energy

Starting from the equation of motion for $G_{ij}(\tau)$ with $\hat{H} = \hat{h} + \hat{V}$, one shows that

$$\boxed{\langle \hat{V} \rangle = -\frac{1}{2} \lim_{\tau \to 0^-} \mathrm{Tr}\left[\left(\mathbf{S} \left(\frac{\partial}{\partial \tau} - \mu \right) + \mathbf{h} \right) \mathbf{G}(\tau) \right].} \tag{1.26}$$

Since $\hat{H} = \hat{h} + \hat{V}$ and $\langle \hat{h} \rangle = \mathrm{Tr}[\mathbf{hP}]$, the total energy is

$$\boxed{\langle \hat{H} \rangle = \mathrm{Tr}\left[\mathbf{hP} - \frac{1}{2} \lim_{\tau \to 0^-} \left(\left(\frac{\partial}{\partial \tau} - \mu \right) \mathbf{S} + \mathbf{h} \right) \mathbf{G}(\tau) \right].} \tag{1.27}$$

Converting the $\tau \to 0^-$ trace to a Matsubara sum and using the inverse Dyson equation, one obtains the frequency-domain total energy

$$\boxed{\langle \hat{H} \rangle = \frac{1}{2} \mathrm{Tr}[(\mathbf{h} + \mathbf{F})\mathbf{P}] + \frac{1}{\beta} \sum_{n=-\infty}^{\infty} e^{-i\omega_n 0^-} \mathrm{Tr}[\mathbf{\Sigma}(i\omega_n)\mathbf{G}(i\omega_n)].} \tag{1.28}$$

Using the conjugation symmetry of real AO quantities, the sum reduces to $n \geq 0$:

$$\boxed{\langle \hat{H} \rangle = \frac{1}{2} \mathrm{Tr}[(\mathbf{h} + \mathbf{F})\mathbf{P}] + \frac{2}{\beta} \mathrm{Re} \sum_{n=0}^{\infty} e^{-i\omega_n 0^-} \mathrm{Tr}[\mathbf{\Sigma}(i\omega_n)\mathbf{G}(i\omega_n)].} \tag{1.29}$$

The equivalent time-domain form is

$$\boxed{\langle \hat{H} \rangle = \frac{1}{2} \mathrm{Tr}[(\mathbf{h} + \mathbf{F})\mathbf{P}] - \int_0^\beta \mathrm{Tr}[\mathbf{\Sigma}(\tau)\mathbf{G}(\beta - \tau)] \, d\tau.} \tag{1.30}$$

1.7 Self-consistent electronic GF2 procedure

Combining the derived equations, we can now perform a self-consistent GF2 loop.

1. Perform an initial Hartree-Fock calculation.
2. Use the output of the HF calculation to set an initial guess for the Fock matrix and density matrix, i.e. $\mathbf{F} = \mathbf{F}_{HF}$ and $\mathbf{P} = \mathbf{P}_{HF}$.
3. Initialize the frequency-domain self-energy on a chosen Matsubara grid: $\mathbf{\Sigma}(i\omega_n) = \mathbf{0}$ for $n = 0, 1, \ldots, N_\omega - 1$.
4. Given $\mathbf{\Sigma}(i\omega_n)$ and $\mathbf{F}$, determine the chemical potential μ such that the electron number constraint holds:

   $$\mathrm{Tr}[\mathbf{PS}] = N_e,$$

   where $\mathbf{P}$ is obtained from the Matsubara summation Eq. (1.17) with $\mathbf{G}(i\omega_n)$ computed via Dyson Eq. (1.21).
5. Solve Dyson's equation on the Matsubara grid to obtain the interacting Green's function:

   $$\mathbf{G}(i\omega_n) = [(\mu + i\omega_n)\mathbf{S} - \mathbf{F} - \mathbf{\Sigma}(i\omega_n)]^{-1}.$$

6. Transform $\mathbf{G}(i\omega_n)$ to imaginary time to obtain $\mathbf{G}(\tau)$ on a grid using the Fourier series consistent with Eq. (1.15).

7. Build the density matrix from the interacting Green's function:

$$\mathbf{P} = \frac{4}{\beta} \mathrm{Re} \sum_{n=0}^{N_\omega - 1} e^{-i\omega_n 0^-} \mathbf{G}(i\omega_n),$$

and enforce $\mathrm{Tr}[\mathbf{PS}] = N_e$ by updating μ (return to step 4 if needed).

8. Update the Fock matrix from $\mathbf{P}$:

$$F_{ij} = h_{ij} + \frac{1}{2} P_{kl} \left(2v_{ijkl} - v_{ilkj}\right)$$

(Eq. (1.22)). Iterate steps 4-8 until $\mathbf{F}$ (or equivalently $\mathbf{P}$) is self-consistent at fixed $\mathbf{\Sigma}(i\omega_n)$.

9. Construct the GF2 self-energy in imaginary time using the second-order expression:

$$\Sigma_{ij}(\tau) = -G_{kl}(\tau) G_{mn}(\tau) G_{pq}(\beta - \tau)\, v_{impk} \left(2v_{jnlq} - v_{jlnq}\right),$$

which yields $\mathbf{\Sigma}(\tau)$ on the imaginary-time grid.

10. Transform $\mathbf{\Sigma}(\tau)$ to $\mathbf{\Sigma}(i\omega_n)$ on the Matsubara grid.

11. Evaluate the GF2 correlation energy from $\mathbf{G}(\tau)$ and $\mathbf{\Sigma}(\tau)$, and form the total energy

$$E_{\mathrm{GF2}} = E_{\mathrm{core}}[\mathbf{P}] + E_{\mathrm{corr}}^{\mathrm{GF2}}[\mathbf{G}, \mathbf{\Sigma}],$$

where $E_{\mathrm{core}}[\mathbf{P}]$ is the usual one-body + mean-field energy (plus E_{nuc}).

12. Check convergence (e.g. change in total energy, and optionally $\|\Delta\Sigma\|$ or $\|\Delta G\|$). If not converged, return to step 4 and repeat until self-consistency is reached.

When the electronic self-energy is evaluated once using the Hartree-Fock Green's function, the MP2 correlation energy is obtained in the zero-temperature limit as

$$E_{\mathrm{MP2}}^{\mathrm{corr}} = -\frac{1}{2} \int_0^\beta d\tau\, \mathrm{Tr}[\mathbf{\Sigma}^{ee}[\mathbf{G}_0](\tau) \mathbf{G}_0(\beta - \tau)], \qquad \beta \to \infty. \tag{1.31}$$

This perturbative expression is distinct from evaluating the coupled self-consistent total-energy expression with all propagators fixed at their mean-field values.

2 QED-GF2

In this derivation, we use the single-mode Pauli-Fierz Hamiltonian, where the one- and two-electron integrals include the dipole self-energy contribution, i.e. $h_{ij} \equiv h_{ij}^{\mathrm{eff}}$ and $v_{ijkl} \equiv v_{ijkl}^{\mathrm{eff}}$. Defining $X \equiv b + b^\dagger$ for convenience, the Hamiltonian is

$$\boxed{\hat{H} = \sum_{ij} h_{ij}^{\mathrm{eff}} a_i^\dagger a_j + \frac{1}{2} \sum_{ijkl} v_{ijkl}^{\mathrm{eff}} a_i^\dagger a_k^\dagger a_l a_j + \omega_c b^\dagger b + \sum_{ij} g_{ij} a_i^\dagger a_j X.} \tag{2.1}$$

The QED-GF2 expansion is built around a mean-field light-matter reference. In the main text this reference is generated by the CS-HF or LF-HF procedure, and its one-body electronic part is denoted by the QED-dressed Fock matrix $\tilde{\mathbf{F}}$. The following derivation first uses the CS partition. The LF interaction tensors and residual vertex are specified in Sec. 2.9. Thus

$$\boxed{\hat{K}_0 = \sum_{ij} \left(\tilde{F}_{ij} - \mu S_{ij}\right) a_i^\dagger a_j + \omega_c b^\dagger b.} \tag{2.2}$$

The residual interaction is

$$\hat{K}_{\text{int}} = \hat{K} - \hat{K}_0 = \hat{V}_{ee}^{\text{res}} + \hat{V}_{eb}^{\text{fluc}} + E_{\text{off}}, \tag{2.3}$$

where E_{off} collects the constant terms of the mean-field partition, $\hat{V}_{ee}^{\text{res}}$ produces the usual GF2 second-Born electronic self-energy and

$$\hat{V}_{eb}^{\text{fluc}} = (\hat{Q} - A)\, \delta X, \qquad \hat{Q} = \sum_{ij\sigma} g_{ij} a_{i\sigma}^\dagger a_{j\sigma}, \quad A = \text{Tr}[\mathbf{gP}]. \tag{2.4}$$

The CS displacement is included in the static reference. Here δX is the remaining fluctuation coordinate after the shift by 2ξ, with $\xi = -A/\omega_c$. We rename $\delta X \to X$ in the following equations. At self-consistency,

$$\boxed{\langle X \rangle = 0.} \tag{2.5}$$

Centering both the electronic coupling operator and the bosonic coordinate removes the static tadpole from the residual perturbation. All static one-body light-matter effects are counted once through $\tilde{\mathbf{F}}$; the self-energies below contain only the dynamical beyond-mean-field corrections.

2.1 Matsubara Green's Functions

The electronic Green's function is

$$\tilde{G}_{ij}(\tau_1, \tau_2) \equiv -\langle \mathcal{T}_\tau a_i(\tau_1) a_j^\dagger(\tau_2) \rangle. \tag{2.6}$$

Similarly, we define a bosonic Green's function D such that

$$D(\tau_1, \tau_2) \equiv -\langle \mathcal{T}_\tau X(\tau_1) X(\tau_2) \rangle. \tag{2.7}$$

For a time-independent equilibrium reference, both depend only on the time difference. The KMS boundary conditions are

$$\tilde{G}_{ij}(\tau + \beta) = -\tilde{G}_{ij}(\tau), \qquad D(\tau + \beta) = D(\tau). \tag{2.8}$$

Therefore

$$\tilde{\mathbf{G}}(i\omega_n) = \int_0^\beta d\tau\, e^{i\omega_n \tau} \tilde{\mathbf{G}}(\tau), \qquad \tilde{\mathbf{G}}(\tau) = \frac{1}{\beta} \sum_n e^{-i\omega_n \tau} \tilde{\mathbf{G}}(i\omega_n), \tag{2.9}$$

with fermionic Matsubara frequencies $\omega_n = (2n+1)\pi/\beta$, and

$$D(i\nu_\ell) = \int_0^\beta d\tau\, e^{i\nu_\ell \tau} D(\tau), \qquad D(\tau) = \frac{1}{\beta} \sum_\ell e^{-i\nu_\ell \tau} D(i\nu_\ell), \tag{2.10}$$

with bosonic Matsubara frequencies $\nu_\ell = 2\pi\ell/\beta$.

2.2 Non-interacting boson propagator

For the single free cavity mode, the bosonic part of the non-interacting grand-canonical Hamiltonian is $\hat{K}_{0,b} = \omega_c b^\dagger b$ with $X \equiv b + b^\dagger$. Evaluating $D_0(\tau) \equiv -\langle \mathcal{T}_\tau X(\tau) X(0) \rangle_0$ using the imaginary-time operators $b(\tau) = e^{-\omega_c \tau} b$ and $b^\dagger(\tau) = e^{+\omega_c \tau} b^\dagger$ and the Bose occupation $n_B(\omega_c) = (e^{\beta\omega_c} - 1)^{-1}$, one obtains for $0 \leq \tau \leq \beta$:

$$\boxed{D_0(\tau) = -\frac{\cosh\left[\omega_c\left(\tau - \frac{\beta}{2}\right)\right]}{\sinh\left(\frac{\beta\omega_c}{2}\right)}.} \tag{2.11}$$

The Fourier transform using bosonic Matsubara frequencies gives

$$\boxed{D_0(i\nu_\ell) = \frac{1}{i\nu_\ell - \omega_c} - \frac{1}{i\nu_\ell + \omega_c} = -\frac{2\omega_c}{\nu_\ell^2 + \omega_c^2}.} \tag{2.12}$$

This is the non-interacting boson propagator used in the QED-GF2 Dyson equations.

2.3 Derivation of the electron-boson self-energy and polarization

We now derive the electron-boson Fan self-energy and the bosonic polarization from the residual electron-boson interaction. In the interaction picture with respect to $\hat{K}_0$, the residual electron-boson term is

$$\hat{V}_{eb}(\tau) = \sum_{mn\sigma} g_{mn} \left[a^\dagger_{m\sigma}(\tau) a_{n\sigma}(\tau) - \langle a^\dagger_{m\sigma} a_{n\sigma}\rangle_0\right] X(\tau), \tag{2.13}$$

where, after the mean-field displacement has been absorbed into the reference, $\langle X \rangle_0 = 0$. The corresponding imaginary-time evolution operator is

$$\hat{\mathcal{S}} = \mathcal{T}_\tau \exp\left[-\int_0^\beta d\tau\, \hat{V}_{eb}(\tau)\right]. \tag{2.14}$$

The subtraction in Eq. (2.13) removes contractions of the electronic operators within the same interaction vertex. In the contractions below, this subtraction is implicit. The same centering is enforced with the correlated density when the propagators are dressed self-consistently.

The elementary Wick contractions are

$$\left\langle \mathcal{T}_\tau a_i(1) a^\dagger_j(2) \right\rangle_0 = -\tilde{G}^{(0)}_{ij}(1,2), \qquad \langle \mathcal{T}_\tau X(1) X(2) \rangle_0 = -D_0(1,2). \tag{2.15}$$

Here $1, 2, \ldots$ denote imaginary-time arguments, and repeated orbital indices are summed.

2.3.1 Electron-boson contribution to the fermionic self-energy

The electron-boson contribution to the electronic Green's function begins at second order because the first-order term contains one unpaired bosonic field and vanishes when $\langle X \rangle_0 = 0$. Expanding the electronic Green's function to second order gives

$$\begin{aligned} \tilde{G}^{(2),eb}_{ij}(1,2) = -\frac{1}{2} \int d3\, d4 \sum_{mnpq} g_{mn} g_{pq} \\ \times \left\langle \mathcal{T}_\tau a_i(1) a^\dagger_j(2) a^\dagger_m(3) a_n(3) X(3) a^\dagger_p(4) a_q(4) X(4) \right\rangle_{0,c}. \end{aligned} \tag{2.16}$$

The subscript c indicates that disconnected pieces are removed by the normalization denominator $\langle\hat{\mathcal{S}}\rangle_0$.

The bosonic part contracts as

$$\langle\mathcal{T}_\tau X(3)X(4)\rangle_0 = -D_0(3,4). \tag{2.17}$$

The connected fermionic Wick contractions that produce the one-particle irreducible Fan insertion are

$$\begin{aligned}&\left\langle\mathcal{T}_\tau a_i(1)a_j^\dagger(2)a_m^\dagger(3)a_n(3)a_p^\dagger(4)a_q(4)\right\rangle_{0,c}^{\text{Fan}}\\ &\quad= -\tilde{G}_{im}^{(0)}(1,3)\tilde{G}_{np}^{(0)}(3,4)\tilde{G}_{qj}^{(0)}(4,2)-\tilde{G}_{ip}^{(0)}(1,4)\tilde{G}_{qm}^{(0)}(4,3)\tilde{G}_{nj}^{(0)}(3,2).\end{aligned} \tag{2.18}$$

The two terms are equivalent under the interchange $3 \leftrightarrow 4$ together with $(m,n) \leftrightarrow (p,q)$, so they cancel the factor of $1/2$ in Eq. (2.16). Therefore

$$\tilde{G}_{ij}^{(2),eb}(1,2) = \int d3\,d4\,\tilde{G}_{im}^{(0)}(1,3)\left[-g_{mn}\tilde{G}_{np}^{(0)}(3,4)g_{pq}D_0(3,4)\right]\tilde{G}_{qj}^{(0)}(4,2). \tag{2.19}$$

Comparing Eq. (2.19) with the Dyson expansion

$$\tilde{G}_{ij}^{(2),eb}(1,2) = \int d3\,d4\,\tilde{G}_{ik}^{(0)}(1,3)\tilde{\Sigma}_{kl}^{eb,(2)}(3,4)\tilde{G}_{lj}^{(0)}(4,2), \tag{2.20}$$

we identify the bare second-order Fan self-energy as

$$\boxed{\tilde{\Sigma}_{ij}^{eb,(2)}(1,2) = -\sum_{kl} g_{ik}\,\tilde{G}_{kl}^{(0)}(1,2)\,g_{lj}\,D_0(1,2).} \tag{2.21}$$

The self-consistent QED-GF2 approximation is obtained by replacing the bare propagators by dressed propagators. Thus,

$$\boxed{\tilde{\Sigma}_{ij}^{eb}(1,2) = -\sum_{kl} g_{ik}\,\tilde{G}_{kl}(1,2)\,g_{lj}\,D(1,2).} \tag{2.22}$$

For an equilibrium system this depends only on $\tau = \tau_1 - \tau_2$. On the principal interval $0 < \tau < \beta$,

$$\boxed{\tilde{\Sigma}_{ij}^{eb}(\tau) = -\sum_{kl} g_{ik}\,\tilde{G}_{kl}(\tau)\,g_{lj}\,D(\tau).} \tag{2.23}$$

Fourier transforming Eq. (2.23) gives

$$\boxed{\tilde{\Sigma}_{ij}^{eb}(i\omega_n) = -\frac{1}{\beta}\sum_{\ell}\sum_{kl} g_{ik}\,\tilde{G}_{kl}(i\omega_n - i\nu_\ell)\,g_{lj}\,D(i\nu_\ell).} \tag{2.24}$$

The corresponding bosonic polarization is

$$\boxed{\Pi(\tau) = -2\sum_{ij}\sum_{kl} g_{ij}\,\tilde{G}_{jk}(\tau)\,g_{kl}\,\tilde{G}_{li}(\beta-\tau), \qquad 0 < \tau < \beta.} \tag{2.25}$$

or equivalently

$$\boxed{\Pi(i\nu_\ell) = \frac{2}{\beta}\sum_{n}\sum_{ij}\sum_{kl} g_{ij}\,\tilde{G}_{jk}(i\omega_n)\,g_{kl}\,\tilde{G}_{li}(i\omega_n - i\nu_\ell).} \tag{2.26}$$

2.4 Dyson equations

The Dyson equations are

$$\tilde{\mathbf{G}}^{-1}(i\omega_n) = \tilde{\mathbf{G}}_0^{-1}(i\omega_n) - \tilde{\mathbf{\Sigma}}(i\omega_n), \qquad D^{-1}(i\nu_\ell) = D_0^{-1}(i\nu_\ell) - \Pi(i\nu_\ell), \tag{2.27}$$

with

$$\tilde{\mathbf{G}}_0(i\omega_n) = \left[(\mu + i\omega_n)\mathbf{S} - \tilde{\mathbf{F}}\right]^{-1}. \tag{2.28}$$

The total fermionic self-energy is

$$\boxed{\tilde{\mathbf{\Sigma}}(i\omega_n) = \tilde{\mathbf{\Sigma}}^{ee}(i\omega_n) + \tilde{\mathbf{\Sigma}}^{eb}(i\omega_n).} \tag{2.29}$$

The electronic Dyson equation in the notation of the main text is therefore

$$\boxed{\tilde{\mathbf{G}}(i\omega_n) = \left[(\mu + i\omega_n)\mathbf{S} - \tilde{\mathbf{F}} - \tilde{\mathbf{\Sigma}}^{ee}(i\omega_n) - \tilde{\mathbf{\Sigma}}^{eb}(i\omega_n)\right]^{-1}.} \tag{2.30}$$

The ordinary GF2 electronic self-energy is evaluated with the QED-dressed propagator and the effective two-electron integrals:

$$\boxed{\tilde{\Sigma}^{ee}_{ij}(\tau) = -\tilde{G}_{kl}(\tau)\tilde{G}_{mn}(\tau)\tilde{G}_{pq}(\beta - \tau)\, v^{\text{eff}}_{impk}\left(2v^{\text{eff}}_{jnlq} - v^{\text{eff}}_{jlnq}\right), \qquad 0 < \tau < \beta.} \tag{2.31}$$

The electron-boson self-energy is

$$\boxed{\tilde{\Sigma}^{eb}_{ij}(\tau) = -\sum_{kl} g_{ik}\tilde{G}_{kl}(\tau)g_{lj}D(\tau), \qquad 0 < \tau < \beta.} \tag{2.32}$$

The bosonic Dyson equation is

$$\boxed{D(i\nu_\ell) = \left[D_0^{-1}(i\nu_\ell) - \Pi(i\nu_\ell)\right]^{-1}.} \tag{2.33}$$

2.5 Total energy of the coupled electron-boson system

As we saw in Eq. (1.30) for a purely electronic Hamiltonian, the total energy is equal to the core energy plus the GF2 correlation energy calculated using the Galitskii-Migdal expression [11]. For a coupled fermion-boson Hamiltonian however, we need the energy resulting from the bosonic part and the fermion-boson interaction to be able to calculate an accurate total energy. This point has been explicitly worked out for electron-boson Green's function theory by Säkkinen *et al.* in Ref. [12]. As shown in their Sec. II.D the equilibrium total energy is written as

$$E = E_e + E_b + E_{eb}, \tag{2.34}$$

and Appendix A derives the corresponding generalized Galitskii-Migdal functional as a function of *both* the fermionic and bosonic propagators. This is the direct electron-phonon analogue of the present electron-photon problem. Consequently, contracting only the total fermionic self-energy with $\tilde{G}$ is not, by itself, the complete energy of the coupled electron-boson system. As described in Ref. [12] both the bosonic energy and the electron-boson interaction energy contain a classical (mean-field) contribution in addition to the fluctuation contribution. In the case of this work, the CS displacement provides the direct analogue here: for a real coherent displacement ξ, the undisplaced coordinate has expectation value 2ξ, so the classical photon and light-matter pieces are $\omega_c\xi^2$ and $2\xi A$, respectively. However, these terms are already contained in the CS mean-field energy and must not be generated a second time. In the CS partition used here, the static coherent

displacement has therefore been absorbed into the QED mean-field reference. It is useful to separate the energy into a static CS contribution and fluctuation contributions,

$$\boxed{E_{\mathrm{QED-GF2}} = E^{\mathrm{CS}}_{\mathrm{stat}}[\mathbf{P}] + E^{\mathrm{corr}}_{ee} + E^{\mathrm{fluc}}_{eb} + E^{\mathrm{fluc}}_{b}.} \tag{2.35}$$

The four terms in the above equation are given below.

2.5.1 Static coherent-state contribution

At the correlated fixed point, the same expectation value is evaluated with the correlated density,

$$A \equiv \mathrm{Tr}[\mathbf{gP}], \qquad \xi = -\frac{A}{\omega_c}, \tag{2.36}$$

where $\mathbf{P}$ is the spin-summed correlated density matrix and ξ is the stationary coherent displacement. With the one- and two-electron dipole-self-energy contributions already included in h^{eff} and v^{eff}, respectively, the static CS energy is evaluated directly as

$$\boxed{\begin{aligned} E^{\mathrm{CS}}_{\mathrm{stat}}[\mathbf{P}] = \mathrm{Tr}[\mathbf{h}^{\mathrm{eff}}\mathbf{P}] + \frac{1}{2}\mathrm{Tr}\left[\left(\mathbf{J}^{\mathrm{eff}} - \frac{1}{2}\mathbf{K}^{\mathrm{eff}}\right)\mathbf{P}\right] \\ + 2\xi A + \omega_c \xi^2 + E_{\mathrm{nuc}}. \end{aligned}} \tag{2.37}$$

Equation (2.37) counts the coherent contribution $2\xi A + \omega_c\xi^2$ exactly once. The fluctuation coordinate used in the following equations is the displaced coordinate satisfying $\langle X \rangle = 0$; hence no static tadpole contribution is to be added again to the dynamical terms.

2.5.2 Electronic correlation contribution

The electron-electron part is the usual restricted GF2 contribution evaluated with the QED-dressed electronic propagator using the Galitskii-Migdal expression,

$$\boxed{C_{ee} \equiv -\int_0^\beta d\tau\, \mathrm{Tr}\left[\tilde{\boldsymbol{\Sigma}}^{ee}(\tau)\tilde{\mathbf{G}}(\beta-\tau)\right], \qquad E^{\mathrm{corr}}_{ee} = C_{ee}.} \tag{2.38}$$

In the coupled energy expression, $\tilde{\mathbf{G}}$ is a one-spin restricted Green's function. The factor $1/2$ of the general spin-resolved two-body Galitskii-Migdal expression is canceled by the two identical closed-shell spin channels, giving the full one-spin trace in Eq. (2.38) [1].

2.5.3 Residual electron-boson interaction energy

We now derive the energy associated with the *residual* linear electron-boson interaction after the coherent displacement has been absorbed into the reference. The derivation follows the equation-of-motion construction used by Ref. [12] for a coupled electron-phonon problem, specialized here to the single-mode CS-QED partition. In Sec. II.D of Ref. [12], the electron-phonon interaction energy is separated into a classical (mean-field) piece and a dynamical piece. In their Matsubara notation, the latter is obtained by contracting the non-Hartree electronic self-energy with the electronic Green's function,

$$E_{ep} - E^{C}_{ep} = -\sum_{pq}\int_0^\beta d\tau\, \left[\Sigma^{M}_{pq}(-\tau) - \Sigma^{M}_{H,pq}(-\tau)\right] G^{M}_{qp}(\tau), \tag{2.39}$$

with no additional factor 1/2. Appendix A of Ref. [12] derives the same result directly from the electronic equation of motion and combines it with the bosonic energy to obtain a generalized Galitskii-Migdal functional depending on both G and D. In the present CS partition, the analogue of E^C_{ep} is the coherent light–matter contribution $2\xi A$ already included in Eq. (2.37), while the Hartree/tadpole contribution is removed from the residual problem by Eq. (2.5). Therefore only the connected Fan part of the self-energy contributes to the residual interaction energy derived below.

To make the spin counting explicit, let us write the electronic coupling operator with spatial-orbital indices i, j and a spin index σ,

$$\hat{Q} = \sum_{\sigma} \sum_{ij} g_{ij}\, a^{\dagger}_{i\sigma} a_{j\sigma}. \tag{2.40}$$

Because the displaced fluctuation coordinate satisfies $\langle X \rangle = 0$, the residual interaction expectation value can be written as the connected mixed electron-boson correlator

$$E^{\text{fluc}}_{eb} \equiv \left\langle (\hat{Q} - A) X \right\rangle = \langle \hat{Q} X \rangle_{\text{conn}}. \tag{2.41}$$

Equation (2.39) gives this connected contribution directly in terms of the electronic self-energy. The Matsubara functions of Ref. [12] carry the contour factor i relative to the convention of Eqs. (2.6) and (2.7); for the fermionic quantities one has $G^M = i\tilde{G}$ and $\Sigma^M = i\tilde{\Sigma}$. Using also the fermionic KMS relation $\tilde{\Sigma}(-\tau) = -\tilde{\Sigma}(\beta - \tau)$ for $0 < \tau < \beta$, Eq. (2.39) becomes [12, 13], in the present convention,

$$\boxed{E^{\text{fluc}}_{eb} = -\sum_{\sigma} \int_0^{\beta} d\tau\, \mathrm{Tr}_e\left[\tilde{\mathbf{\Sigma}}^{eb}_{\sigma}(\tau) \tilde{\mathbf{G}}_{\sigma}(\beta - \tau)\right],} \tag{2.42}$$

where Tr_e denotes a trace over spatial electronic orbitals only.

For a closed-shell reference,

$$\tilde{\mathbf{G}}_{\uparrow} = \tilde{\mathbf{G}}_{\downarrow} \equiv \tilde{\mathbf{G}}, \qquad \tilde{\mathbf{\Sigma}}^{eb}_{\uparrow} = \tilde{\mathbf{\Sigma}}^{eb}_{\downarrow} \equiv \tilde{\mathbf{\Sigma}}^{eb}, \tag{2.43}$$

so the explicit spin sum in Eq. (2.42) gives a factor of two. Defining the one-spin contraction

$$C_{eb} \equiv -\int_0^{\beta} d\tau\, \mathrm{Tr}_e\left[\tilde{\mathbf{\Sigma}}^{eb}(\tau) \tilde{\mathbf{G}}(\beta - \tau)\right], \tag{2.44}$$

we obtain

$$\boxed{E^{\text{fluc}}_{eb} = -2 \int_0^{\beta} d\tau\, \mathrm{Tr}_e\left[\tilde{\mathbf{\Sigma}}^{eb}(\tau) \tilde{\mathbf{G}}(\beta - \tau)\right] \equiv 2C_{eb}.} \tag{2.45}$$

The same interaction energy follows independently from the bosonic polarization, providing a second derivation that uses only the QED-GF2 self-energy definitions. For a single mode, Eq. (2.32) gives

$$\begin{aligned} \mathrm{Tr}_e\left[\tilde{\mathbf{\Sigma}}^{eb}(\tau) \tilde{\mathbf{G}}(\beta - \tau)\right] &= -D(\tau) \sum_{ijkl} g_{ik} \tilde{G}_{kl}(\tau) g_{lj} \tilde{G}_{ji}(\beta - \tau) \\ &= -D(\tau) \mathrm{Tr}_e\left[\mathbf{g} \tilde{\mathbf{G}}(\tau) \mathbf{g} \tilde{\mathbf{G}}(\beta - \tau)\right]. \end{aligned} \tag{2.46}$$

Define the electronic bubble

$$\mathcal{B}(\tau) \equiv \mathrm{Tr}_e\left[\mathbf{g} \tilde{\mathbf{G}}(\tau) \mathbf{g} \tilde{\mathbf{G}}(\beta - \tau)\right]. \tag{2.47}$$

The factor of two in the closed-shell polarization, Eq. (2.25), gives

$$\Pi(\tau) = -2\mathcal{B}(\tau), \tag{2.48}$$

and hence Eq. (2.46) becomes the pointwise identity

$$\boxed{\mathrm{Tr}_e\left[\tilde{\mathbf{\Sigma}}^{eb}(\tau)\tilde{\mathbf{G}}(\beta-\tau)\right] = \frac{1}{2}\Pi(\tau)D(\tau).} \tag{2.49}$$

Substitution into Eq. (2.44) yields

$$\boxed{C_{eb} = -\frac{1}{2}\int_0^\beta d\tau\,\Pi(\tau)D(\tau), \qquad E_{eb}^{\mathrm{fluc}} = -\int_0^\beta d\tau\,\Pi(\tau)D(\tau).} \tag{2.50}$$

Thus the fermionic and bosonic routes evaluate the same residual electron-boson interaction energy, similar to the electron-phonon energy decomposition of Ref. [12], where the electronic equation of motion produces the $\Sigma^{eb}G$ form, while the bosonic response produces the equivalent ΠD form.

Using the Fourier conventions of Eqs. (2.10) and (2.26), the same result may be written as

$$\boxed{E_{eb}^{\mathrm{fluc}} = -\frac{1}{\beta}\sum_\ell \mathrm{Tr}_b\left[\mathbf{\Pi}^T(i\nu_\ell)\mathbf{D}(-i\nu_\ell)\right].} \tag{2.51}$$

For a real single-mode equilibrium problem, $D(-i\nu_\ell) = D(i\nu_\ell)$ and the analogous evenness holds for Π, so Eq. (2.51) reduces to the usual scalar Matsubara sum. Equations (2.45), (2.50), and (2.51) therefore provide mutually equivalent implementations of the residual electron-boson interaction energy and a stringent internal consistency test at self-consistency.

The equality of the $\Sigma^{eb}G$ and ΠD contractions follows algebraically when both kernels are constructed from the same G, D, and residual vertex. It is therefore an internal consistency relation, but does not by itself establish convergence of the coupled Dyson equations.

2.5.4 Bosonic fluctuation energy

The electron-phonon formulation of Ref. [12] evaluates the bosonic energy from the equal-time bosonic propagator in a coordinate-momentum representation, in the present work however, we propagate only the displacement correlator $D(\tau)$.

For a single cavity mode, let us introduce the conjugate dimensionless quadrature

$$Y \equiv i(b^\dagger - b), \qquad [X, Y] = 2i. \tag{2.52}$$

With the photon zero-point energy omitted, as in Eq. (2.1),

$$\hat{H}_b = \omega_c b^\dagger b = \frac{\omega_c}{4}\left(X^2 + Y^2 - 2\right). \tag{2.53}$$

Hence

$$E_b^{\mathrm{fluc}} = \frac{\omega_c}{4}\left(\langle X^2\rangle + \langle Y^2\rangle - 2\right). \tag{2.54}$$

The displacement propagator gives

$$\langle X^2\rangle = -D(0^-). \tag{2.55}$$

The remaining momentum-quadrature expectation value can be eliminated by the stationary bosonic virial identity [14]. Define

$$\hat{\mathcal{A}} = \frac{1}{4}(XY + YX). \tag{2.56}$$

For an equilibrium state, $\langle[\hat{H}, \hat{\mathcal{A}}]\rangle = 0$. Since the residual coupling is $\hat{V}_{eb}^{\text{fluc}} = (\hat{Q} - A)X$, with $\hat{Q}$ defined in Eq. (2.40), direct evaluation of the commutator gives

$$\boxed{\frac{\omega_c}{2}\left(\langle Y^2\rangle - \langle X^2\rangle\right) = \langle(\hat{Q} - A)X\rangle = E_{eb}^{\text{fluc}}.} \tag{2.57}$$

Substituting Eqs. (2.55) and (2.57) into Eq. (2.54) yields an expression involving only quantities available in the QED-GF2 calculation,

$$\boxed{E_b^{\text{fluc}} = -\frac{\omega_c}{2}\left[D(0^-) + 1\right] + \frac{1}{2}E_{eb}^{\text{fluc}}.} \tag{2.58}$$

This form contains no photon zero-point contribution. As a check, at zero electron–boson coupling $D \to D_0$ and $E_{eb}^{\text{fluc}} \to 0$. Since

$$D_0(0^-) = -\coth\left(\frac{\beta\omega_c}{2}\right), \tag{2.59}$$

Eq. (2.58) reduces to $E_b = \omega_c n_B(\omega_c)$, which vanishes as $\beta \to \infty$.

For numerical evaluation of the equal-time value, the analytic bare contribution can be separated from the dressed correction,

$$\boxed{D(0^-) = D_0(0^-) + \frac{1}{\beta}\sum_{\ell}\left[D(i\nu_\ell) - D_0(i\nu_\ell)\right].} \tag{2.60}$$

The difference $D - D_0$ decays more rapidly at large $|\nu_\ell|$ than either propagator separately. Equation (2.60) is exact for the infinite Matsubara sum. On a finite grid, convergence of the remaining sum over $D - D_0$ must still be checked.

2.5.5 Total CS-GF2 energy expression

Collecting Eqs. (2.37), (2.38), (2.45), and (2.58), the coupled CS-GF2 energy is

$$\boxed{E_{\text{CS-GF2}} = E_{\text{stat}}^{\text{CS}}[\mathbf{P}] + C_{ee} + E_{eb}^{\text{fluc}} + E_b^{\text{fluc}}.} \tag{2.61}$$

Equivalently, because $E_{eb}^{\text{fluc}} = 2C_{eb}$,

$$\boxed{E_{\text{CS-GF2}} = E_{\text{stat}}^{\text{CS}}[\mathbf{P}] + C_{ee} + 3C_{eb} - \frac{\omega_c}{2}\left[D(0^-) + 1\right].} \tag{2.62}$$

Equation (2.61) differs qualitatively from applying the ordinary electronic Galitskii-Migdal expression to $\tilde{\Sigma}^{ee} + \tilde{\Sigma}^{eb}$. The latter contains only a fermionic contraction and therefore does not separately account for the bosonic fluctuation energy [12].

2.6 Validation of the total-energy expression using an exactly solvable model

To independently validate the coupled electron-boson total-energy expression, we consider the following two-site, two-electron Hamiltonian. The calculations in this subsection are designed specifically to verify the energy expressions derived above. In particular, the direct Hamiltonian expectation values are compared with independent reconstructions obtained from the exact electronic and bosonic Green's functions. These calculations therefore validate the energy identities and their implementation, rather than the accuracy of the approximate CS-GF2 self-energies.

$$\hat{H} = \hat{H}_1 + \hat{H}_{ee} + \hat{H}_b + \hat{H}_{eb}, \tag{2.63}$$

where

$$\hat{H}_1 = -t \sum_{\sigma} \left(c^{\dagger}_{1\sigma} c_{2\sigma} + c^{\dagger}_{2\sigma} c_{1\sigma} \right), \tag{2.64}$$

$$\hat{H}_{ee} = U \sum_{i=1}^{2} n_{i\uparrow} n_{i\downarrow}, \tag{2.65}$$

$$\hat{H}_b = \omega_c b^{\dagger} b, \tag{2.66}$$

$$\hat{H}_{eb} = g(n_1 - n_2)(b + b^{\dagger}). \tag{2.67}$$

This Hubbard-Holstein model contains the same electronic one-body, electronic two-body, bosonic, and linear electron-boson structure required to test the coupled energy expression. It is not a molecular Pauli-Fierz Hamiltonian, and no molecular dipole self-energy is included in this model calculation.

We diagonalized the Hamiltonian in the complete two-electron space constructed from four spin orbitals and a truncated photon-number basis. The photon states n = $0, \ldots, N_{\text{ph}}$ were retained, with $N_{\text{ph}} = 24$ used for the reported calculations. The individual energy contributions were evaluated directly as

$$E_1^{\text{ED}} = \langle \Psi_0 | \hat{H}_1 | \Psi_0 \rangle, \tag{2.68}$$

$$E_{ee}^{\text{ED}} = \langle \Psi_0 | \hat{H}_{ee} | \Psi_0 \rangle, \tag{2.69}$$

$$E_{eb}^{\text{ED}} = \langle \Psi_0 | \hat{H}_{eb} | \Psi_0 \rangle, \tag{2.70}$$

$$E_b^{\text{ED}} = \langle \Psi_0 | \hat{H}_b | \Psi_0 \rangle. \tag{2.71}$$

Their sum gives a direct evaluation of the exact total energy.

The exact electronic Green's function was constructed from the electron-addition and electron-removal Lehmann representations using the one-electron and three-electron eigenstates. To separate the electronic and electron-boson self-energy contributions, we introduced the equation-of-motion Green's functions

$$F_{ij}^{\alpha}(z) = \left\langle\!\!\left\langle [c_i, \hat{H}_\alpha]; c_j^{\dagger} \right\rangle\!\!\right\rangle_z, \qquad \alpha = ee, eb, \tag{2.72}$$

from which the corresponding self-energy components were obtained as

$$\boldsymbol{\Sigma}^U(z) = \mathbf{F}^{ee}(z)\mathbf{G}^{-1}(z), \tag{2.73}$$

$$\boldsymbol{\Sigma}^{eb}(z) = \mathbf{F}^{eb}(z)\mathbf{G}^{-1}(z), \tag{2.74}$$

$$\boldsymbol{\Sigma}^{ee}(z) = \boldsymbol{\Sigma}^U(z) - \boldsymbol{\Sigma}^{\text{HF}}, \tag{2.75}$$

Here, $\boldsymbol{\Sigma}^U$ is the full self-energy associated with the Hubbard interaction, while $\Sigma_{ij}^{\text{HF}} = U \langle \hat{n}_{i\bar{\sigma}} \rangle \delta_{ij}$. The subtraction in the last line removes the static Hartree-Fock contribution already included in E_{ee}^{HF}, so that C_{ee} contains only the electronic correlation contribution. This construction is independent of the direct expectation values of the corresponding Hamiltonian terms.

Within the spin-restricted formulation, the energy contributions reconstructed from the exact Green's functions are

$$E_1^G = 2\text{Tr}[\mathbf{h}\boldsymbol{\rho}], \tag{2.76}$$

$$E_{ee}^G = E_{ee}^{\text{HF}} + C_{ee}, \tag{2.77}$$

$$E_{eb}^G = 2C_{eb}, \tag{2.78}$$

$$E_b^G = -\frac{\omega_c}{2} \left[D(0^-) + 1 \right] + C_{eb}. \tag{2.79}$$

where

$$C_\alpha = -\int_0^\beta d\tau \, \mathrm{Tr}\left[\mathbf{\Sigma}^\alpha(\tau)\mathbf{G}(\beta-\tau)\right]. \tag{2.80}$$

The electron-boson interaction energy was also reconstructed independently from the exact bosonic polarization as

$$E_{eb}^G = -\int_0^\beta d\tau \, \Pi(\tau)D(\tau). \tag{2.81}$$

The corresponding total energy is

$$E^G = E_{\mathrm{stat}} + C_{ee} + 3C_{eb} - \frac{\omega_c}{2}\left[D(0^-) + 1\right]. \tag{2.82}$$

Table 2.6 compares the direct and reconstructed energy contributions for the interacting cases with $U/t = 2$. Both the electronic correlation and photon contributions are nonzero in these calculations.

Table S1: Direct Hamiltonian expectation values and Green's-function reconstructions for the two-site Hubbard-Holstein model with $t = \omega_c = 1$ and $U/t = 2$. All energies are reported in units of t.

	$g = 0.2$			$g = 0.5$		
Contribution	Direct	Green's function	$\lvert\Delta\rvert$	Direct	Green's function	$\lvert\Delta\rvert$
E_1	−1.7910750862	−1.7910750862	4.44×10^{-16}	−1.7959616178	−1.7959616178	2.22×10^{-16}
E_{ee}	0.5634703894	0.5634703894	3.33×10^{-16}	0.6357901174	0.6357901174	6.66×10^{-16}
E_{eb}	−0.0218685194	−0.0218685194	2.43×10^{-17}	−0.1836375360	−0.1836375360	1.94×10^{-16}
E_b	0.0027224384	0.0027224384	1.58×10^{-16}	0.0308407875	0.0308407875	3.23×10^{-16}
E	−1.2467507779	−1.2467507779	0.00	−1.3129682489	−1.3129682489	1.33×10^{-15}

We repeated the comparison for $U/t = 0$ and 2, $g/t = 0$, 0.2, and 0.5, with $\omega_c/t = 1$. Across these six calculations, the maximum absolute error in any individual contribution reconstructed from the fermionic self-energy contractions was $8.9\times10^{-16}t$, and the maximum absolute error in the total energy was $4.0\times10^{-15}t$. The independent reconstruction using the ΠD expression gave maximum errors of $9.9 \times 10^{-14}t$ for the electron-boson interaction energy and $1.0 \times 10^{-13}t$ for the total energy.

We successively increased the photon-number cutoff from $N_{\mathrm{ph}} = 8$ to 16 and 24. At $N_{\mathrm{ph}} = 24$, the probability in the highest retained photon state was below 6.0×10^{-28} for all reported calculations. The imaginary-frequency contractions were evaluated using 240 quadrature points and a frequency cutoff of $200t$. We repeated the calculations with twice the quadrature order and twice the frequency cutoff, which did not change the reported conclusions.

As a separate implementation check, we passed the same one-electron integrals, two-electron integrals, photon frequency, and electron-boson vertex directly to the production CS-GF2 solver. All six calculations converged. The electron-boson interaction energies obtained from the production $\Sigma^{eb}G$ and ΠD contractions agreed within $2.2 \times 10^{-16}t$. This confirms that the production GF2 calculations use the same coupled energy decomposition validated above. This check verifies the implementation of the energy expression, but it does not imply that the second-Born and Fan self-energies used in CS-GF2 are exact for this model. Their accuracy is assessed separately below through comparison with exact diagonalization.

2.7 CS-GF2 benchmark for the two-site Hubbard-Holstein model

We next use the same model to assess the accuracy of the approximate CS-GF2 method. This comparison is distinct from the validation in Sec. 2.6. In the preceding subsection, exact propagators, exact channel self-energies, and direct Hamiltonian expectation values were used to verify the coupled energy identities. Here, the electronic self-energy, Fan electron-boson self-energy, and bubble polarization are evaluated self-consistently using the production CS-GF2 implementation and are compared with exact diagonalization.

The benchmark uses two electrons in four spin orbitals with $t = \omega_c = 1$. We consider $U/t = 0.1$ and 6 and vary the electron-boson coupling over the range of $g/t = 0, 0.05, \ldots, 0.50$. In the site basis, the model quantities supplied to the production solver are

$$\mathbf{S} = \mathbf{I}, \qquad \mathbf{h} = \begin{pmatrix} 0 & -t \\ -t & 0 \end{pmatrix}, \qquad (11|11) = (22|22) = U, \qquad \mathbf{g} = \begin{pmatrix} g & 0 \\ 0 & -g \end{pmatrix}. \tag{2.83}$$

Exact diagonalization was performed in the canonical two-electron space using bosonic occupations $n = 0, \ldots, 24$. The bosonic-space cutoff was checked by repeating each exact calculation with occupations $n = 0, \ldots, 16$. We also performed a grand-canonical exact-diagonalization calculation at the same inverse temperature as CS-GF2, adjusting the chemical potential to give $\langle N \rangle = 2$, to verify that the residual finite-temperature contribution is negligible relative to the reported ground-state comparison.

The CS-HF reference was converged independently from bonding, site-1, and site-2 initial density matrices. The lowest converged stationary solution was used for CS-MP2 and CS-GF2. Because we used the same site energy, we get zero coherent displacement, and consequently the relative energy $E_{\mathrm{CS-HF}}(g) - E_{\mathrm{CS-HF}}(0)$ is identically zero. CS-HF is therefore omitted from the corresponding main-text panels.

The CS-GF2 calculations used $\beta t = 200$, 300 electronic imaginary-time points, 300 explicitly sampled positive fermionic Matsubara frequencies, a 10,000-point positive-frequency grid for the frequency-to-time transformations, and 50 Legendre terms. The model integrals and CS-HF reference were supplied directly to the production CS-GF2 driver. The electronic and bosonic self-energy kernels, Matsubara transformations, Dyson equations, coupled total-energy expression, and convergence tests were unchanged from those used for the molecular calculations.

Table S2: Absolute energies and signed errors relative to exact diagonalization for representative points from the two-site Hubbard-Holstein benchmark. All quantities are reported in units of t, and the signed errors are defined as $E_{\mathrm{method}} - E_{\mathrm{ED}}$.

U/t	g/t	E_{ED}/t	$E_{\mathrm{CS-MP2}}/t$	$E_{\mathrm{CS-GF2}}/t$	$\Delta E_{\mathrm{CS-MP2}}/t$	$\Delta E_{\mathrm{CS-GF2}}/t$
0.1	0.00	−1.9506249024	−1.9506250000	−1.9506269017	−0.0000000976	−0.0000019993
0.1	0.25	−1.9927832294	−1.9922916667	−1.9935388264	+0.0004915627	−0.0007555971
0.1	0.50	−2.1563179153	−2.1172916667	−2.1734335449	+0.0390262487	−0.0171156295
6.0	0.00	−0.6055512755	−1.2500000000	−0.1715382841	−0.6444487245	+0.4340129914
6.0	0.25	−0.6083485228	−1.2916666667	−0.1925538617	−0.6833181439	+0.4157946611
6.0	0.50	−0.6172231336	−1.4166666667	−0.2670278322	−0.7994435331	+0.3501953014

Table S3: Maximum numerical checks over the eleven coupling values for each U/t in the two-site Hubbard-Holstein benchmark. Energy-based quantities are reported in units of t. The quantities R_e and R_b are the electronic and bosonic Dyson residuals defined in Eq. (2.85).

U/t	ED cutoff change	Exact thermal shift	R_e	R_b	Maximum GF2 iterations
0.1	2.22×10^{-16}	3.11×10^{-15}	2.27×10^{-6}	3.83×10^{-9}	85
6.0	7.77×10^{-16}	4.11×10^{-15}	3.22×10^{-7}	3.37×10^{-9}	107

All 22 CS-GF2 calculations converged according to the convergence criteria. The exact-diagonalization cutoff changes and finite-temperature exact shifts are negligible on the scale of the plotted energies. Both Dyson residuals were monitored independently, and the electron-boson interaction energies evaluated from the $\Sigma^{eb}G$ and ΠD contractions agreed to within $1.11 \times 10^{-16} t$ over the complete benchmark.

2.8 Practical self-consistency loop

A compact implementation loop is:

1. Start from a converged QED mean-field solution, defining $\tilde{\mathbf{F}}$, $\tilde{E}$, and an initial $\tilde{\mathbf{G}}$ with $\tilde{\mathbf{\Sigma}} = 0$.
2. Given $\tilde{\mathbf{G}}$, construct the electronic GF2 self-energy $\tilde{\mathbf{\Sigma}}^{ee}$ from Eq. (2.31).
3. Given $\tilde{\mathbf{G}}$, construct the bosonic polarization Π from Eqs. (2.25) and (2.26) and update D from the bosonic Dyson equation Eq. (2.33).
4. Given $\tilde{\mathbf{G}}$ and D, construct the electron-boson self-energy $\tilde{\mathbf{\Sigma}}^{eb}$ from Eqs. (2.32) and (2.24).
5. Form $\tilde{\mathbf{\Sigma}} = \tilde{\mathbf{\Sigma}}^{ee} + \tilde{\mathbf{\Sigma}}^{eb}$ from Eq. (2.29) and update $\tilde{\mathbf{G}}$ from the electronic Dyson equation Eq. (2.30).
6. Update the density matrix from Eq. (1.17) and rebuild the QED-dressed Fock matrix, update the stationary coherent displacement, and adjust the chemical potential to maintain the required electron number.
7. Evaluate C_{ee} from Eq. (2.38), E_{eb}^{fluc} from Eq. (2.45), the photon fluctuation energy from Eq. (2.58), and the total energy from Eq. (2.61). Iterate until the electronic and bosonic Dyson residuals and the total energy are converged.

To check convergence of the coupled equations, the electronic and bosonic Dyson residuals can be evaluated as

$$\mathbf{R}_e(i\omega_n) = \left[(\mu + i\omega_n)\mathbf{S} - \tilde{\mathbf{F}} - \tilde{\mathbf{\Sigma}}^{ee}[G](i\omega_n) - \tilde{\mathbf{\Sigma}}^{eb}[G, D](i\omega_n)\right] \tilde{\mathbf{G}}(i\omega_n) - \mathbf{I}, \tag{2.84}$$

$$R_b(i\nu_\ell) = D^{-1}(i\nu_\ell) - D_0^{-1}(i\nu_\ell) + \Pi[G](i\nu_\ell). \tag{2.85}$$

Here the Fock matrix and self-energies are rebuilt from the current density and propagators. Both residuals must be checked, since a small change in the total energy alone does not establish convergence of the coupled equations.

2.9 Projected Lang–Firsov GF2 (LF-GF2)

In the present work, LF-GF2 denotes a *projected* Lang–Firsov formulation. Vacuum expectation values of the LF displacement operators define the transformed electronic one- and two-body terms, while the residual explicit linear electron–boson vertex is retained and treated dynamically. The second-order LF reference below is constructed from this projected Hamiltonian to specify the perturbative comparison with LF-GF2.

Electronic correlation is treated self-consistently with a second-Born/GF2 self-energy constructed from the projected LF interaction tensor, while the residual electron–boson interaction is treated using the same Fan approximation employed in CS-GF2. The LF parameters are optimized using the *fully self-consistent correlated LF-GF2 energy*, rather than the static LF reference energy alone.

2.9.1 Fixed orthonormal basis for the LF transformation

The diagonal LF transformation is basis dependent. We therefore define it in a fixed orthonormal one-electron basis obtained from symmetric orthogonalization of the AO basis,

$$\mathbf{X} = \mathbf{S}^{-1/2}, \tag{2.86}$$

where $\mathbf{S}$ is the AO overlap matrix. One- and two-electron quantities are transformed according to

$$h_{pq} = X_{\mu p} h_{\mu\nu} X_{\nu q}, \tag{2.87}$$

$$g_{pq} = X_{\mu p} g_{\mu\nu} X_{\nu q}, \tag{2.88}$$

$$v_{pqrs} = X_{\mu p} X_{\nu q} X_{\kappa r} X_{\lambda s} v_{\mu\nu\kappa\lambda}. \tag{2.89}$$

Repeated AO indices are summed. All LF equations below are expressed in this fixed orthonormal basis, and the basis labels are suppressed for notational simplicity. The orbital basis itself is not optimized together with the LF parameters.

For the single-mode case, the LF transformation is parameterized by one real displacement parameter λ_p for every orthonormal orbital and by a coherent displacement ξ. We denote the full set of orbital-dependent LF parameters by

$$\boldsymbol{\lambda} = (\lambda_1, \lambda_2, \dots, \lambda_{N_{\text{orb}}}). \tag{2.90}$$

The Hamiltonian used in the LF transformation is the Pauli–Fierz Hamiltonian after inclusion of the dipole self-energy contribution, written schematically as

$$\hat{H}_{\text{PF}} = \hat{H}_{\text{e}}^{\text{PF}} + \omega_c b^\dagger b + \sum_{pq} g_{pq} a_p^\dagger a_q (b + b^\dagger), \tag{2.91}$$

where ω_c is the cavity frequency and g_{pq} denotes the electron–photon coupling matrix in the LF basis.

2.9.2 Franck–Condon factors and vacuum projection

Under the diagonal LF transformation, fermionic operators acquire photon-displacement operators. Their vacuum expectation values produce the one- and two-electron Franck–Condon factors [15]

$$F_{pq}^{(1)} = \exp\left[-\frac{1}{2}(\lambda_q - \lambda_p)^2\right], \tag{2.92}$$

and

$$F_{pqrs}^{(2)} = \exp\left[-\frac{1}{2}\left(\lambda_q - \lambda_p + \lambda_s - \lambda_r\right)^2\right]. \tag{2.93}$$

Projecting the cloud operators that multiply the purely electronic terms onto the photon vacuum gives the projected LF one-electron matrix

$$\begin{aligned} h_{pq}^{\text{LF}} = {} & F_{pq}^{(1)} \left[h_{pq}^{\text{PF}} + 2\xi g_{pq} - (\lambda_p + \lambda_q) g_{pq}\right] \\ & + \delta_{pq}\left(\omega_c \lambda_p^2 - 2\omega_c \xi \lambda_p\right), \end{aligned} \tag{2.94}$$

where h^{PF} is the Pauli–Fierz one-electron matrix in the same orthonormal basis.

The corresponding projected two-electron interaction is

$$\begin{aligned} v_{pqrs}^{\text{LF}} = {} & F_{pqrs}^{(2)} v_{pqrs}^{\text{PF}} + 2\omega_c \lambda_p \lambda_r \delta_{pq} \delta_{rs} \\ & - 2F_{pq}^{(1)} g_{pq} \lambda_r \delta_{rs} - 2F_{rs}^{(1)} g_{rs} \lambda_p \delta_{pq}. \end{aligned} \tag{2.95}$$

The last two terms in Eq. (2.95) are the pair-exchanged contributions generated by the LF transformation. The projected tensor retains the Hermitian pair-exchange structure required by the transformed Hamiltonian, but it does not in general retain the separate within-pair permutation symmetries of ordinary Coulomb integrals. Consequently, the exchange contractions must preserve the literal index ordering of v^{LF}.

For a spin-restricted calculation with spin-summed density matrix **P**, the LF Fock matrix is

$$F_{pq}^{\mathrm{LF}} = h_{pq}^{\mathrm{LF}} + J_{pq}^{\mathrm{LF}} - \frac{1}{2}K_{pq}^{\mathrm{LF}}, \tag{2.96}$$

with

$$J_{pq}^{\mathrm{LF}} = \sum_{rs} v_{pqrs}^{\mathrm{LF}} P_{rs}, \tag{2.97}$$

$$K_{pq}^{\mathrm{LF}} = \sum_{rs} v_{psrq}^{\mathrm{LF}} P_{rs}. \tag{2.98}$$

The use of v_{psrq}^{LF} in the exchange term is essential for the reduced-symmetry transformed interaction; replacing it by a symmetry-related permutation valid for ordinary Coulomb integrals is not equivalent after the LF transformation.

The corresponding static LF energy is

$$E_{\mathrm{stat}}^{\mathrm{LF}} = \sum_{pq} h_{pq}^{\mathrm{LF}} P_{qp} + \frac{1}{2}\sum_{pq}\left(J_{pq}^{\mathrm{LF}} - \frac{1}{2}K_{pq}^{\mathrm{LF}}\right) P_{qp} + \omega_c \xi^2 + E_{\mathrm{nuc}}. \tag{2.99}$$

For LF-HF, Eq. (2.99) is evaluated with the mean-field density. In LF-GF2, the same static expression is evaluated with the correlated density matrix.

2.9.3 Residual electron–boson vertex

The vacuum projection is applied to the LF cloud operators multiplying the electronic terms, but the remaining explicit linear photon fluctuation is retained. Defining the one-body vacuum cloud factor

$$C_{pq}^{(1)} = \exp\left[-\frac{1}{2}(\lambda_q - \lambda_p)^2\right], \tag{2.100}$$

the residual projected electron–boson vertex is [15]

$$\boxed{g_{pq}^{\mathrm{LF}} = C_{pq}^{(1)} g_{pq} - \omega_c \lambda_p \delta_{pq}.} \tag{2.101}$$

Since $C_{pp}^{(1)} = 1$,

$$g_{pp}^{\mathrm{LF}} = g_{pp} - \omega_c \lambda_p. \tag{2.102}$$

Thus, the LF transformation absorbs part of the original electron–boson coupling into the projected electronic Hamiltonian, while the remaining dynamical linear coupling is described explicitly by g^{LF}.

The residual vertex is used in the Fan self-energy and photon polarization,

$$\Sigma^{\mathrm{eb,LF}}(\tau) = -g^{\mathrm{LF}} G(\tau) g^{\mathrm{LF}} D(\tau), \tag{2.103}$$

$$\Pi^{\mathrm{LF}}(\tau) = -2\,\mathrm{Tr}\left[g^{\mathrm{LF}} G(\tau) g^{\mathrm{LF}} G(\beta - \tau)\right]. \tag{2.104}$$

In the coherent-state limit, $\boldsymbol{\lambda} = \mathbf{0}$, $C_{pq}^{(1)} = 1$, and $g_{pq}^{\mathrm{LF}} = g_{pq}$.

To make the projected interaction content explicit, the Hamiltonian used in the LF-GF2 procedure can be written as

$$
\begin{aligned}
\hat{H}^{\mathrm{LF}}_{\mathrm{proj}} = E_{\mathrm{nuc}} + \omega_c \xi^2 + \sum_{pq\sigma} h^{\mathrm{LF}}_{pq} a^\dagger_{p\sigma} a_{q\sigma} \\
+ \frac{1}{2} \sum_{pqrs\sigma\sigma'} v^{\mathrm{LF}}_{pqrs} a^\dagger_{p\sigma} a^\dagger_{r\sigma'} a_{s\sigma'} a_{q\sigma} \\
+ \omega_c b^\dagger b + (\hat{Q}^{\mathrm{LF}} + \omega_c \xi) X,
\end{aligned}
\tag{2.105}
$$

where $\hat{Q}^{\mathrm{LF}} = \sum_{pq\sigma} g^{\mathrm{LF}}_{pq} a^\dagger_{p\sigma} a_{q\sigma}$. The stationary coherent shift gives $\omega_c \xi = -A^{\mathrm{LF}}$, with $A^{\mathrm{LF}} = \mathrm{Tr}[\mathbf{g}^{\mathrm{LF}} \mathbf{P}]$. The residual interaction is therefore $(\hat{Q}^{\mathrm{LF}} - A^{\mathrm{LF}})X$. The vacuum averages define the projected electronic integrals, and the explicit linear interaction remains in the coupled Hamiltonian.

2.9.4 Reduced-symmetry electronic GF2 self-energy

Because v^{LF} does not possess the full permutation symmetry of ordinary Coulomb integrals, the electronic second-Born self-energy must also retain the literal ordering of the LF interaction vertices. In the fixed orthonormal LF basis, the restricted electronic self-energy is

$$
\begin{aligned}
\Sigma^{ee,\mathrm{LF}}_{ij}(\tau) = - \sum_{klmnpq} v^{\mathrm{LF}}_{ikqm} G_{kl}(\tau) G_{mn}(\tau) G_{pq}(\beta - \tau) \\
\times \left[2 v^{\mathrm{LF}}_{pnjl} - v^{\mathrm{LF}}_{pljn} \right], \qquad 0 < \tau < \beta.
\end{aligned}
\tag{2.106}
$$

For an ordinary Coulomb-symmetric interaction tensor, Eq. (2.106) reduces to the standard restricted second-Born/GF2 expression. All Green's functions entering Eq. (2.106) are the dressed Green's functions obtained from the projected LF-GF2 Dyson equation.

2.9.5 Stationary coherent displacement and correlated LF optimization

For a fixed $\boldsymbol{\lambda}$ and density matrix $\mathbf{P}$, the coherent displacement is eliminated analytically by imposing stationarity of the projected LF energy. With the residual vertex of Eq. (2.101), this gives

$$
\boxed{\xi(\boldsymbol{\lambda}; \mathbf{P}) = -\frac{1}{\omega_c} \sum_{pq} g^{\mathrm{LF}}_{pq} P_{qp}.}
\tag{2.107}
$$

Thus ξ is not treated as an independent numerical optimization variable. In LF-GF2, for each trial $\boldsymbol{\lambda}$ the coupled electronic and bosonic Dyson equations are converged self-consistently, Eq. (2.107) is enforced with the correlated density matrix, and the fully converged LF-GF2 total energy is used as the objective for the outer optimization of $\boldsymbol{\lambda}$.

The correlated LF-GF2 total energy is evaluated using the same decomposition as the CS expression,

$$
\begin{aligned}
E_{\mathrm{LF-GF2}} = E^{\mathrm{LF}}_{\mathrm{stat}} + C^{\mathrm{LF}}_{ee} + 3 C^{\mathrm{LF}}_{eb} - \frac{\omega_c}{2} [D(0^-) + 1], \\
C^{\mathrm{LF}}_{\alpha} = - \int_0^\beta d\tau \, \mathrm{Tr} \left[\boldsymbol{\Sigma}^{\alpha,\mathrm{LF}}(\tau) \mathbf{G}(\beta - \tau) \right], \qquad \alpha = ee, eb.
\end{aligned}
\tag{2.108}
$$

Here $E^{\mathrm{LF}}_{\mathrm{stat}}$ is given by Eq. (2.99). The electronic self-energy uses v^{LF}, and both the Fan self-energy and polarization use g^{LF}. The coherent photon contribution is included once in the static energy. At $\boldsymbol{\lambda} = \mathbf{0}$, the projected integrals, residual vertex, and static energy reduce to their CS counterparts.

2.9.6 Projected LF-MP2 as the perturbative counterpart of projected LF-GF2

To make the perturbative comparison internally consistent, we define projected LF-MP2 from the *same* projected LF Hamiltonian used in LF-GF2. At a fixed LF reference $(\boldsymbol{\lambda}, \xi)$, let i, j denote occupied LF-HF orbitals, a, b virtual LF-HF orbitals, and ε_p the corresponding orbital energies. The zero-photon electronic matrix element is

$$V^{\mathrm{LF}}_{iajb} = \sum_{pqrs} C_{pi} C_{qa} C_{rj} C_{sb} v^{\mathrm{LF}}_{pqrs}, \tag{2.109}$$

and the purely electronic second-order contribution is

$$E^{(2),\mathrm{LF}}_{ee,0\gamma} = -\sum_{ijab} \frac{V^{\mathrm{LF}}_{iajb}\left(2V^{\mathrm{LF}}_{iajb} - V^{\mathrm{LF}}_{ibja}\right)}{\varepsilon_a + \varepsilon_b - \varepsilon_i - \varepsilon_j}. \tag{2.110}$$

The residual linear vertex retained explicitly in projected LF-GF2 contributes, at second order,

$$E^{(2),\mathrm{LF}}_{eb,1\gamma} = -2\sum_{ia} \frac{\left|g^{\mathrm{LF}}_{ia}\right|^2}{\varepsilon_a - \varepsilon_i + \omega_c}, \qquad g^{\mathrm{LF}}_{ia} = \sum_{pq} C_{pi} g^{\mathrm{LF}}_{pq} C_{qa}. \tag{2.111}$$

A possible pure one-photon contribution proportional to the expectation value of the residual linear coupling vanishes when Eq. (2.107) is satisfied. Therefore the projected LF-MP2 energy used as the direct perturbative counterpart of projected LF-GF2 is

$$\boxed{E_{\text{proj-LFMP2}} = E^{\mathrm{LF}}_{\mathrm{stat}} + E^{(2),\mathrm{LF}}_{ee,0\gamma} + E^{(2),\mathrm{LF}}_{eb,1\gamma}}. \tag{2.112}$$

Equation (2.112) gives the projected second-order energy at fixed $(\boldsymbol{\lambda}, \xi)$ using the LF-HF electronic reference and the free photon propagator. Subsequent Dyson iterations dress the electronic and bosonic propagators self-consistently and give the LF-GF2 energy of Eq. (2.108).

2.9.7 Projected LF-GF2 self-consistency procedure

The projected LF-GF2 calculation is organized as a nested self-consistent procedure. For a given trial LF displacement vector $\boldsymbol{\lambda}$, the orbital-dependent LF parameters are held fixed while the electronic and bosonic Dyson equations are converged self-consistently. The coherent displacement ξ, in contrast, is not an independent optimization variable and is updated analytically from the correlated density matrix at every GF2 iteration according to Eq. (2.107). Only after convergence of this fixed-$\boldsymbol{\lambda}$ coupled problem is the resulting LF-GF2 total energy used to update $\boldsymbol{\lambda}$.

For a fixed trial vector $\boldsymbol{\lambda}$, the inner projected LF-GF2 self-consistency cycle proceeds as follows:

1. Construct the projected LF one-electron matrix h^{LF}, reduced-symmetry two-electron interaction v^{LF}, and residual electron–boson vertex g^{LF} from Eqs. (2.94), (2.95), and (2.101), respectively.

2. Using the current correlated density matrix $\mathbf{P}$, evaluate the stationary coherent displacement

$$\xi = -\frac{1}{\omega_c} \sum_{pq} g^{\mathrm{LF}}_{pq} P_{qp}, \tag{2.113}$$

   and rebuild the projected LF Hamiltonian and LF Fock matrix using Eqs. (2.96) and (2.98).

3. Construct the corresponding mean-field reference electronic Green's function $\mathbf{G}_0(i\omega_n)$ from the LF Fock matrix and adjust the chemical potential so that the required electron number is reproduced.

4. Using the current dressed electronic Green's function $\mathbf{G}$, evaluate the reduced-symmetry electronic second-Born self-energy $\boldsymbol{\Sigma}^{ee,\mathrm{LF}}$ from Eq. (2.106).

5. Using $\mathbf{G}$ and the residual vertex g^{LF}, evaluate the bosonic polarization Π, solve the bosonic Dyson equation for the dressed photon propagator D, and construct the Fan electron–boson self-energy $\boldsymbol{\Sigma}^{eb,\mathrm{LF}}$.

6. Form the total correlation self-energy

$$\boldsymbol{\Sigma}^{\mathrm{LF}} = \boldsymbol{\Sigma}^{ee,\mathrm{LF}} + \boldsymbol{\Sigma}^{eb,\mathrm{LF}}, \tag{2.114}$$

and solve the electronic Dyson equation to obtain an updated dressed Green's function $\mathbf{G}$.

7. Update the correlated density matrix from the dressed Green's function and recompute the chemical potential, LF Fock matrix, and stationary coherent displacement ξ.

8. Evaluate the total energy from Eq. (2.108) and repeat steps 2–7 until the coupled electronic–bosonic fixed point is converged with respect to the total energy, particle number, Fock matrix, electronic self-energy, bosonic propagator and polarization, both Dyson residuals, and the stationary coherent displacement.

For each fixed $\boldsymbol{\lambda}$, this procedure defines the fully self-consistent projected LF-GF2 energy

$$E^{\mathrm{SC}}_{\mathrm{LF-GF2}}(\boldsymbol{\lambda}). \tag{2.115}$$

The LF displacement vector is then optimized in an outer loop using this converged correlated energy as the objective. Numerical optimization is carried out using the Limited-memory Broyden–Fletcher–Goldfarb–Shanno (L-BFGS) method. Nearby trial $\boldsymbol{\lambda}$ vectors are evaluated by rebuilding the transformed Hamiltonian and reconverging the complete coupled GF2 fixed point; previously converged correlated quantities may be used only as initial guesses for the nearby fixed-point solution. Thus, $\boldsymbol{\lambda}$ is never varied inside an unconverged GF2 Dyson iteration. The final parameters satisfy

$$\boldsymbol{\lambda}^{\star} = \arg\min_{\boldsymbol{\lambda}} E^{\mathrm{SC}}_{\mathrm{LF-GF2}}(\boldsymbol{\lambda}), \tag{2.116}$$

2.9.8 Initialization, restarts, and local branch stability

For a new molecular calculation, the CS-HF density is used to initialize the electronic density, and the initial LF displacement vector is set to $\boldsymbol{\lambda} = \mathbf{0}$. During the first LF-HF iteration, the static LF objective was minimized from five reproducible trial vectors, including the incoming vector, the zero vector, and three displaced vectors. The displaced vectors were generated using a fixed random seed and a characteristic scale

$$s_{\lambda} = \frac{\max_{pq} \left| g^{\mathrm{PF}}_{pq} \right|}{\omega_c}. \tag{2.117}$$

The solution with the lowest converged static LF energy was retained. Subsequent LF-HF iterations were initialized using the displacement vector from the preceding iteration. The correlated LF-GF2 optimization was initialized from the converged LF-HF solution. For every trial displacement vector $\boldsymbol{\lambda}$, the electronic and bosonic fixed point was reconverged before the LF-GF2 energy was returned to the outer optimization. Previously converged correlated quantities could be used as initial guesses for nearby displacement vectors, but the energy objective was evaluated only after reconvergence of the complete coupled problem.

We additionally assessed the local stability of every reported optimized LF-GF2 solution. Starting from the selected solution $\boldsymbol{\lambda}^\star$, ten displaced initial vectors were constructed as

$$\boldsymbol{\lambda}_0^{(k)} = \boldsymbol{\lambda}^\star + r_\lambda \mathbf{u}_k, \qquad r_\lambda = 10^{-3}, \qquad \|\mathbf{u}_k\|_2 = 1, \tag{2.118}$$

where the directions $\mathbf{u}_k$ were independently generated using a fixed pseudorandom seed. Each displaced vector was used to initiate an independent outer LF optimization, with the complete electronic and bosonic fixed point reconverged at every evaluation of the correlated energy. Defining

$$\Delta E_k = E^{\mathrm{SC}}_{\mathrm{LF-GF2}}\left(\boldsymbol{\lambda}^{(k)}_{\mathrm{final}}\right) - E^{\mathrm{SC}}_{\mathrm{LF-GF2}}\left(\boldsymbol{\lambda}^\star\right), \tag{2.119}$$

none of the ten restarted optimizations at any reported LF-GF2 point lowered the correlated energy by more than $10^{-6}\ E_h$. The selected solutions are therefore locally stable with respect to the tested displacement and energy resolutions.

The projected LF-MP2 energy is defined by Eq. (2.112), using the same projected electronic interaction and residual linear vertex. The LF-GF2 energy instead uses the converged electronic and bosonic propagators in Eq. (2.108).

3 Convergence Results

The present GF2 implementation uses the finite-temperature Matsubara representation as a numerical route to the ground-state limit. All production GF2 calculations were performed at a single finite inverse temperature of $\beta = 200\ E_h^{-1}$, without extrapolation to $\beta \to \infty$. The electronic Green's function was represented using 300 explicitly sampled positive fermionic Matsubara frequencies, with a dense low-frequency region followed by a logarithmically spaced high-frequency region. These quantities were interpolated onto a 10,000-point positive-frequency grid for the frequency-to-time transformations. The electronic imaginary-time quantities were represented on a 300-point Chebyshev grid, and the self-energy was transformed using a 50-term Legendre representation.

The CS and LF transformations are state-independent unitary transformations. The electronic state multiplied by the bosonic vacuum is used to define the ground-state mean-field reference, while the subsequent coupled Green's-function equations are evaluated in the finite-temperature Matsubara representation.

3.1 β Convergence

To assess convergence toward the low-temperature limit, we varied β while scaling N_ω^{full} and N_τ approximately proportionally with β. Since $\omega_n = (2n+1)\pi/\beta$, this maintains an approximately constant high-frequency window as β is increased. The sparse Matsubara grid ($N_\omega^{\mathrm{sparse}} = 300$) and Legendre order ($N_{\mathrm{Leg}} = 50$) were kept fixed.

Table S4: Convergence of the CS-GF2 total energy of H_2 with respect to β. Absolute energy differences are reported relative to the production value at $\beta = 200\ E_h^{-1}$.

β (E_h^{-1})	$E(R = 0.741$ Å$)$ (E_h)	$\lvert\Delta E\rvert$ (E_h)	$\lvert\Delta E\rvert$ (kcal mol^{-1})	$E(R = 3.000$ Å$)$ (E_h)	$\lvert\Delta E\rvert$ (E_h)	$\lvert\Delta E\rvert$ (kcal mol^{-1})
50	-1.152886641242	1.8463×10^{-4}	0.116	-0.920073339175	1.4897×10^{-3}	0.935
100	-1.153183356660	1.1209×10^{-4}	0.070	-0.921393419186	1.6961×10^{-4}	0.106
200	-1.153071267203	0	0	-0.921563024746	0	0
300	-1.152849397605	2.2187×10^{-4}	0.139	-0.921480118645	8.2906×10^{-5}	0.052

At the stretched geometry, increasing β from the production value of 200 to 300 E_h^{-1} changes the energy by only $8.29 \times 10^{-5}\ E_h$ (0.052 kcal mol^{-1}). At equilibrium, all values between $\beta = 50$ and 300 E_h^{-1} remain within $2.22 \times 10^{-4}\ E_h$ (0.139 kcal mol^{-1}) of the production result.

The largest stretched-geometry deviation occurs at $\beta = 50\ E_h^{-1}$. For $\beta \geq 100\ E_h^{-1}$, the deviation is at most 0.106 kcal mol^{-1}.

3.2 Matsubara Grid Convergence

Grid convergence was tested independently at fixed $\beta = 200\ E_h^{-1}$ by varying the Matsubara-frequency, imaginary-time, and Legendre representations.

Table S5: Convergence of the CS-GF2 total energy for stretched H_2 ($R = 3.000$ Å) with respect to the numerical Matsubara representation. Absolute energy differences are reported relative to the production-grid result.

Grid	$N_\omega^{\mathrm{sparse}}$	N_ω^{full}	N_τ	N_{Leg}	$E_{\mathrm{CS-GF2}}$ (E_h)	$\lvert\Delta E\rvert$ (E_h)	$\lvert\Delta E\rvert$ (kcal mol^{-1})
Coarse	200	7500	200	40	-0.921501081180	6.1944×10^{-5}	0.039
Production	300	10000	300	50	-0.921563024746	0	0
Fine	450	15000	450	70	-0.921520138363	4.2886×10^{-5}	0.027

The coarse and fine grids differ from the production result by only 0.039 and 0.027 kcal mol^{-1}, respectively, demonstrating that the production energy is well converged with respect to the numerical grid representation.

It should be noted that all of the deviations observed in tables above, are below the commonly used threshold of chemical accuracy (1 kcal mol^{-1}). These deviations measure numerical stability relative to the production calculation, rather than the error relative to an exact reference.

4 Optimized Geometries

All geometries were optimized at the levels of theory specified below using Psi4 [16].

$$CH_2{=}CHOH \rightleftharpoons CH_3CHO \quad \text{(vinyl alcohol} \rightleftharpoons \text{acetaldehyde)}$$

Atom	x (Å)	y (Å)	z (Å)
C	-0.432046	0.142303	0.000000
C	-0.470777	-1.206548	0.000000
O	0.725703	0.947700	0.000000
H	-1.313201	0.775639	0.000000
H	0.429084	-1.821581	0.000000
H	-1.424818	-1.724962	0.000000
H	1.541259	0.401997	0.000000

Table S6: Enol geometry (MP2/6-311G)

Atom	x (Å)	y (Å)	z (Å)
C	-0.309057	-0.529932	0.056067
C	1.109158	-0.529904	0.124980
O	-0.757447	0.673526	-0.317086
H	-1.036781	-1.311171	0.266026
H	1.648279	-1.179341	0.812459
H	1.613734	-0.382457	-0.837028
H	0.541154	0.931376	-0.105416

Table S7: Transition state geometry (MP2/6-311G, one imaginary frequency)

Atom	x (Å)	y (Å)	z (Å)
C	-0.357742	0.272724	0.000000
C	-0.348207	-1.241007	0.000000
O	0.681684	0.984697	0.000000
H	-1.353600	0.751977	0.000000
H	0.686589	-1.601564	0.000000
H	-0.873101	-1.624537	0.887338
H	-0.873101	-1.624537	-0.887338

Table S8: Keto geometry (MP2/6-311G)

References

[1] Daniel Neuhauser, Roi Baer, and Dominika Zgid. Stochastic self-consistent second-order green's function method for correlation energies of large electronic systems. *Journal of chemical theory and computation*, 13(11):5396–5403, 2017.

[2] Tyler Y Takeshita, Wenjie Dou, Daniel GA Smith, Wibe A de Jong, Roi Baer, Daniel Neuhauser, and Eran Rabani. Stochastic resolution of identity second-order matsubara green's function theory. *The Journal of chemical physics*, 151(4), 2019.

[3] Alexander A Rusakov and Dominika Zgid. Self-consistent second-order green's function perturbation theory for periodic systems. *The Journal of chemical physics*, 144(5), 2016.

[4] Jordan J Phillips and Dominika Zgid. Communication: The description of strong correlation within self-consistent green's function second-order perturbation theory. *The Journal of chemical physics*, 140(24), 2014.

[5] Péter R Surján. Second quantization for nonorthogonal orbitals. In *Second Quantized Approach to Quantum Chemistry: An Elementary Introduction*, pages 103–113. Springer, 1989.

[6] Kristian S Thygesen. Electron transport through an interacting region: The case of a nonorthogonal basis set. *Physical Review B—Condensed Matter and Materials Physics*, 73(3):035309, 2006.

[7] Rudolf Haag, Nicolaas Marinus Hugenholtz, and Marinus Winnink. On the equilibrium states in quantum statistical mechanics. *Communications in Mathematical Physics*, 5(3):215–236, 1967.

[8] Ryogo Kubo. Statistical-mechanical theory of irreversible processes. i. general theory and simple applications to magnetic and conduction problems. *Journal of the physical society of Japan*, 12(6):570–586, 1957.

[9] Paul C Martin and Julian Schwinger. Theory of many-particle systems. i. *Physical Review*, 115(6):1342, 1959.

[10] Clemens Carel Johannes Roothaan. New developments in molecular orbital theory. *Reviews of modern physics*, 23(2):69, 1951.

[11] V M Galitskii and A B Migdal. Number 1 july. *J. Exptl. Theoret. Phys. (U.S.S.R.)*, 34:139–150, 1958.

[12] Niko Säkkinen, Yang Peng, Heiko Appel, and Robert van Leeuwen. Many-body green's function theory for electron-phonon interactions: Ground state properties of the holstein dimer. *The Journal of Chemical Physics*, 143(23):234101, 2015.

[13] Gianluca Stefanucci and Robert Van Leeuwen. *Nonequilibrium many-body theory of quantum systems: a modern introduction*. Cambridge University Press, 2013.

[14] Iris Theophilou, Markus Penz, Michael Ruggenthaler, and Angel Rubio. Virial relations for electrons coupled to quantum field modes. *Journal of Chemical Theory and Computation*, 16(10):6236, 2020.

[15] Zhi Hao Cui, Arkajit Mandal, and David R. Reichman. Variational lang–firsov approach plus møller–plesset perturbation theory with applications to ab initio polariton chemistry. *Journal of Chemical Theory and Computation*, 20:1143–1156, 2 2024.

[16] Daniel G. A. Smith, Lori A. Burns, Andrew C. Simmonett, Robert M. Parrish, Matthew C. Schieber, Raimondas Galvelis, Peter Kraus, Holger Kruse, Roberto Di Remigio, Asem Alenaizan, Andrew M. James, Susi Lehtola, Jonathon P. Misiewicz, Maximilian Scheurer, Robert A. Shaw, Jeffrey B. Schriber, Yi Xie, Zachary L. Glick, Dominic A. Sirianni, Joseph Senan O'Brien, Jonathan M. Waldrop, Ashutosh Kumar, Edward G. Hohenstein, Benjamin P. Pritchard, Bernard R. Brooks, III Schaefer, Henry F., Alexander Yu. Sokolov, Konrad Patkowski, III DePrince, A. Eugene, Uğur Bozkaya, Rollin A. King, Francesco A. Evangelista, Justin M. Turney, T. Daniel Crawford, and C. David Sherrill. Psi4 1.4: Open-source software for high-throughput quantum chemistry. *The Journal of Chemical Physics*, 152(18):184108, 05 2020.
